# Towards calibration-invariant spectroscopy using deep learning

**DOI:** 10.1038/s41598-019-38482-1

**Published:** 2019-02-14

**Authors:** M. Chatzidakis, G. A. Botton

**Affiliations:** 1 Department of Materials Science and Engineering, McMaster University, 1280 Main Street West, Hamilton, ON L9H 4L7 Canada; 2 Canadian Center for Electron Microscopy, McMaster University, 1280 Main Street West, Hamilton, ON L8S 4M1 Canada

## Abstract

The interaction between matter and electromagnetic radiation provides a rich understanding of what the matter is composed of and how it can be quantified using spectrometers. In many cases, however, the calibration of the spectrometer changes as a function of time (such as in electron spectrometers), or the absolute calibration may be different between different instruments. Calibration differences cause difficulties in comparing the absolute position of measured emission or absorption peaks between different instruments and even different measurements taken at different times on the same instrument. Present methods of avoiding this issue involve manual feature extraction of the original signal or qualitative analysis. Here we propose automated feature extraction using deep convolutional neural networks to determine the class of compound given only the shape of the spectrum. We classify three unique electronic environments of manganese (being relevant to many battery materials applications) in electron energy loss spectroscopy using 2001 spectra we collected in addition to testing on spectra from different instruments. We test a variety of commonly used neural network architectures found in the literature and propose a new fully convolutional architecture with improved translation-invariance which is immune to calibration differences.

## Introduction

A trained human spectroscopist is able to look at an unknown spectrum, which can be thought of as energy-series data, overlay a proposed candidate reference spectrum and determine (qualitatively) if there is a match. The human brain can perform this task with ease despite translational shifts in the spectrum or varying levels of noise between the reference spectrum and acquired spectrum. The rigor of matching unknown spectra to references can be improved using generalized linear models, fitting procedures, or cross-correlation functions but great difficulty arises with these methods in situations with high noise or translational shifts. There is a need to develop new generalizable and automated methods which can remove qualitative interpretation in spectroscopy analysis. Qualitative interpretation in spectroscopy adds bias to the analysis which includes any overlaying of reference spectra, manual peak shifting, and manual feature selection such as using the full-width half-maximum of peaks, or the intensity ratio between peaks. In addition to these issues, many present methods of quantification/identification require reference standards from the same instrument or from instruments with the same calibration which severely limits the amount of data available to a human spectroscopist. In this study, we apply advances in statistical learning algorithms (also called machine learning, or narrow artificial intelligence) to better identify important characteristics of a spectrum.

Domain experts have used feature engineering in the past to develop useful predictor variables (also known as features) which can be used to differentiate spectra. Such features include metrics like the width of peaks, ratio of peaks and distance between peaks. Feature engineering is the manual process of a domain expert performing dimensionality reduction by using domain knowledge to isolate key pieces of information from the original data. This is in contrast to using statistical learning algorithms which are able to find features automatically by aggregating statistics across large datasets. These algorithms learn to find features automatically by viewing many examples of spectra and deciding which pieces of the spectra are the most useful for differentiating between signals. Statistical learning methods in spectroscopy have been slowly percolating the literature for the last couple decades. Gallagher and Deacon, in a pioneering study in 2002, used single layer dense neural networks to predict experimental X-ray spectra for the automated classification of minerals^1^. Timoshenko *et al*. used multi-layer dense neural networks to predict the coordination numbers in metallic nanoparticles given a full simulated x-ray absorption spectrum^2^ and Zheng *et al*. used an ensemble-learned matching scheme to characterize simulated x-ray absorption spectra generated from the Materials Project where one of the steps explicitly involves peak shifting^3^. Carey *et al*. used unsupervised methods such as nearest neighbor clustering approaches and studied the effect of preprocessing on the classification of Raman spectra^4^. Lopez-Reyes *et al*. developed unsupervised (principal component analysis) and supervised methods (dense neural networks) to classify minerals on the ExoMars rover with Raman spectroscopy^5^.

For the classification of the entire Raman spectroscopy database RRUFF, Liu *et al*. used a convolutional neural network feature extractor^6^. Their methodology was two-fold. First a convolutional neural network feature extractor, then for classification, a dense neural network were used. They compared these results to other common machine learning classifiers such as *boosting*, *random forest*, and *support vector machines*. To teach the network to understand small translational (chemical) shifts, they used data augmentation with small random crops/shifts of the spectra.

Convolutional neural networks have not been used presently in electron spectroscopy. Yedra *et al*. created a script in Digital Micrograph (a software environment for acquiring and processing electron energy loss spectroscopy (EELS) data and images), named *Oxide Wizard*, to differentiate the oxidation states for various metal oxides in EELS^7^. Using domain knowledge, they engineer features such as the ratios of peaks, the full width at half maximum (FWHM) of peaks and the distances between oxygen and the metal edges. Zhang *et al*. used a multiple linear least-squares technique to determine the valence of Mn in EELS^8^. With reference spectra, they fit a generalized linear model using known reference spectra. Tan *et al*. also showed a comparison between common methods in EELS to determine the oxidation state, such as least-squares fitting and feature engineering using peak-ratios. They determined that different methods are ideal for different transition metals^9^.

We develop here a challenging dataset to be used as a test-case to probe the effectiveness and generalizability of convolutional neural networks in spectroscopy classification. As a model system, we investigated the valence identification of Mn, this being relevant in many fields of materials research, particularly interesting in the study of valence in battery materials. We acquired 2001 EELS spectra for Mn^2+^, Mn^3+^, and Mn^4+^ to be used as a model training and model validation dataset and we also digitize 31 reference spectra for Mn^2+^, Mn^3+^, and Mn^4+^ from published articles to be used as a test-set. We test three architectures: a densely connected neural network, a convolutional neural network feature extractor connected with a densely connected neural network and finally a fully convolutional neural network without any dense connections.

## Results

Two key benchmarks have been developed which were found to predict performance on the out-of-distribution test set. The first benchmark is to manually translate the validation-set spectra. This was found to approximate the effect of calibration and chemical shift. The second was by adding and removing noise from the data by modeling the noise distribution using combinations of principal components and measuring the resulting change in accuracy.

Three types of neural network architectures are explored in this study. The dense network has 11,000 weights, the convolutional and dense network has 1100 weights and the fully convolutional architecture has 650 weights. The dense neural network is similar to the dense network used in Gallagher and Deacon’s study^1^. More recent advances in neural networks were added to increase performance such as: neuron dropout to prevent overfitting^10^, batch normalization between hidden layers for activation normalization^11^, rectified linear units (ReLU) as activation function for the hidden units, and a softmax activation function for the output neurons. The second architecture is a convolutional neural network feature extractor attached to a densely connected neural network which is similar to the one used by Liu *et al*.^6^. The convolutional neural network architecture was inspired by Liu *et al*.^6^ and the choice for the number of convolutional filters and layers was inspired by the feature extractors used in the ImageNet competition like the well-established AlexNet and VGG16 architectures^12,13^. The third architecture has the same convolutional neural network architecture as previously described, but it is attached to a custom classification architecture inspired by the MobileNet and SqueezeNet architectures^14,15^.

A full description of the neural architectures can be found in the Methods section.

**Figure 1:**
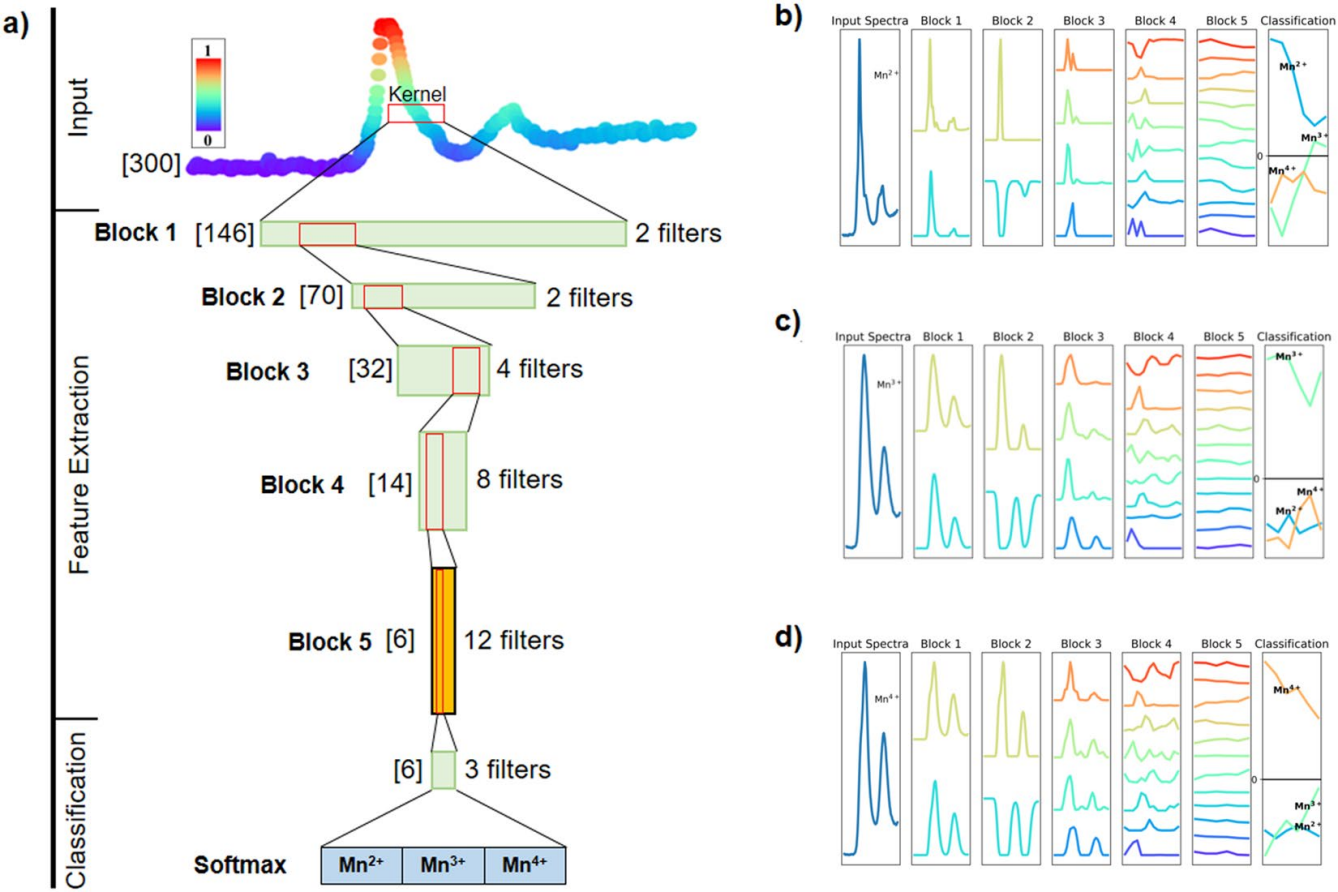
(**a**) Schematic diagram of the neural network graph of the fully convolutional neural network. Bracketed quantities are the length of the spectrum after pooling. The red window is the kernel size of the convolution (see Methods for window sizes). (**b–d**) Activation outputs per feature extraction block and classification block averaged over all training examples of Mn^2+^, Mn^3+^, and Mn^4+^ respectively. The input spectra shown are the average of all spectra in each valence state. The output shown for the Classification block is the output after the final length-1 depth-wise 1D convolution operation (but before global average pooling).

The graph of the fully convolutional neural network architecture can be found in Fig. 1. The feature extraction architecture is also the same used as the convolutional and dense containing network. The output of each operation can be found in Fig. 1b–d for the fully trained network visualized on the training set.

In Fig. 1a, the input spectrum is passed through the 5 successive feature extraction blocks where each block contains a convolution, batch-normalization and down-sampling (1D average pooling). The output of the 5th block (orange) is considered to be a representation of the original input data that is optimized for discrimination (i.e. discriminative features). These features are then used to classify the valence of the inputted spectrum using the single classification block which contains: dropout, convolution (kernel size, 1), global average pooling, and softmax. A description of every operation and relevant parameters can be found in the Methods section.

### Data Collection

A total of 2001 electron energy-loss spectra of Mn^2+^, Mn^3+^, and Mn^4+^ were acquired using a FEI Titan transmission electron microscope in a variety of conditions. This includes 448 Mn^2+^ spectra, 765 Mn^3+^ spectra, and 788 Mn^4+^ spectra cropped between 635 and 665 eV (300 bins at a dispersion of 0.1 eV/bin). The microscope parameters and spectrum image preprocessing steps can be found in the Methods section.

**Figure 2:**
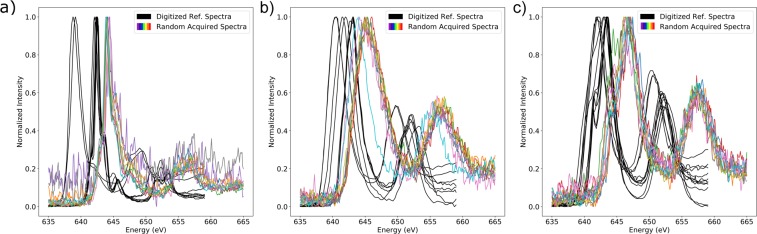
(**a**) 12 digitized reference spectra of various Mn^2+^ compounds taken from published articles^8,9,16^ (black). 12 randomly selected MnO (Mn^2+^) spectra acquired for this study. (**b**) 10 digitized reference spectra of various Mn^3+^ compounds taken from published articles (black). 10 randomly selected Mn_2_O_3_ (Mn^3+^) spectra acquired for this study. (**c**) 9 digitized reference spectra of various Mn^4+^ compounds taken from published articles (black). 10 randomly selected MnO_2_ (Mn^4+^) spectra acquired for this study. All digitized spectra shown are taken from digitizing the figures in Zhang *et al*., (reprinted from American Mineralogist 95 1741–1746, (2010) with permission from the Mineralogical Society of America), Tan *et al*. (reprinted from Ultramicroscopy 116, 24–33, (2012) with permission of Elsevier) and Garvie and Craven (reprinted from Phys. Chem. Miner. 21, 191–206 (1994), with Permission from Springer Nature)^8,9,16^.

To obtain spectra from a wide variety of instruments and resolutions to prove generalizability, reference spectra were digitized from three studies by Garvie *et al*., Zhang *et al*. and Tan *et al*.^8,9,16^. These spectra were not used for model training and are instead used as a withheld test-set representing signals that are well outside of the distribution of the acquired data. They are of significantly higher resolution and the differences in instrument calibration is clear, with the onsets of the peaks being different as shown in Fig. 2. The energy range of the digitized spectra is between 635 and 658 eV due to many of them being cropped for their respective publications.

A qualitative inspection of Fig. 2 highlights that Mn^2+^ is narrower than both Mn^3+^ and Mn^4+^. In addition, Mn^4+^ can be differentiated from Mn^3+^ by a small shoulder located on the low energy side of the larger peak.

A flow-chart describing the pipe-line of going from acquired data to a functioning model can be found in the Supporting Information (Figure S1).

### Model Training

Stratified 10-fold cross-validation was used for model validation and to estimate the error of the validation set. The acquired Mn dataset was divided into 10 roughly equal folds where each class is stratified. 9 folds (i.e. 90% of the data) was used for finding model parameters while the last fold was used for calculating withheld-set validation accuracy. This was repeated for every fold in the model to produce 10 models trained on different partitions of the data.

In batches of 128 randomly selected augmented training spectra at a time, the spectra are non-linearly transformed over all edges and nodes in the directed graph to produce predicted class probabilities $p({y}_{k}^{(i)}|z,\theta )$ for all possible classes. The cost function being minimized *C*(*θ*) is the categorical cross-entropy loss between the true one-hot encoded label ${y}_{k}^{(i)}$ and the predicted class probability $p({y}_{k}^{(i)}|x,\theta )$ calculated over all *N* training examples in the batch and for all possible *K* classes.

$$C(\theta )=-\sum _{i=1}^{N}\sum _{k=1}^{K}\,{y}_{k}^{(i)}\,\mathrm{log}\,p({y}_{k}^{(i)}|x,\theta )$$

The error from the cost function can be back-propagated backwards using the chain-rule through each weight and bias in the network to assign blame as to which parameters were unhelpful in the classification. With this scheme, the weights and biases can be nudged in a direction that minimizes error using stochastic gradient descent (SGD). The specific SGD algorithm used was the adaptive moment estimation optimizer, the Adam optimizer commonly used for the training of neural networks (default parameters)^17^.

### Model Validation – Effect of translation

Depending on the calibration of the spectrometer, it is possible for the peaks in the measured spectrum to be translated. In electron spectroscopy, the electronic environment of the atom being measured can also cause translational shifts (i.e. different compounds with the same oxidation state can be shifted). Because of this, it is crucial that an electron spectroscopy classifier must understand the shape of the spectra and not simply memorize the absolute onset of the peak.

**Figure 3:**
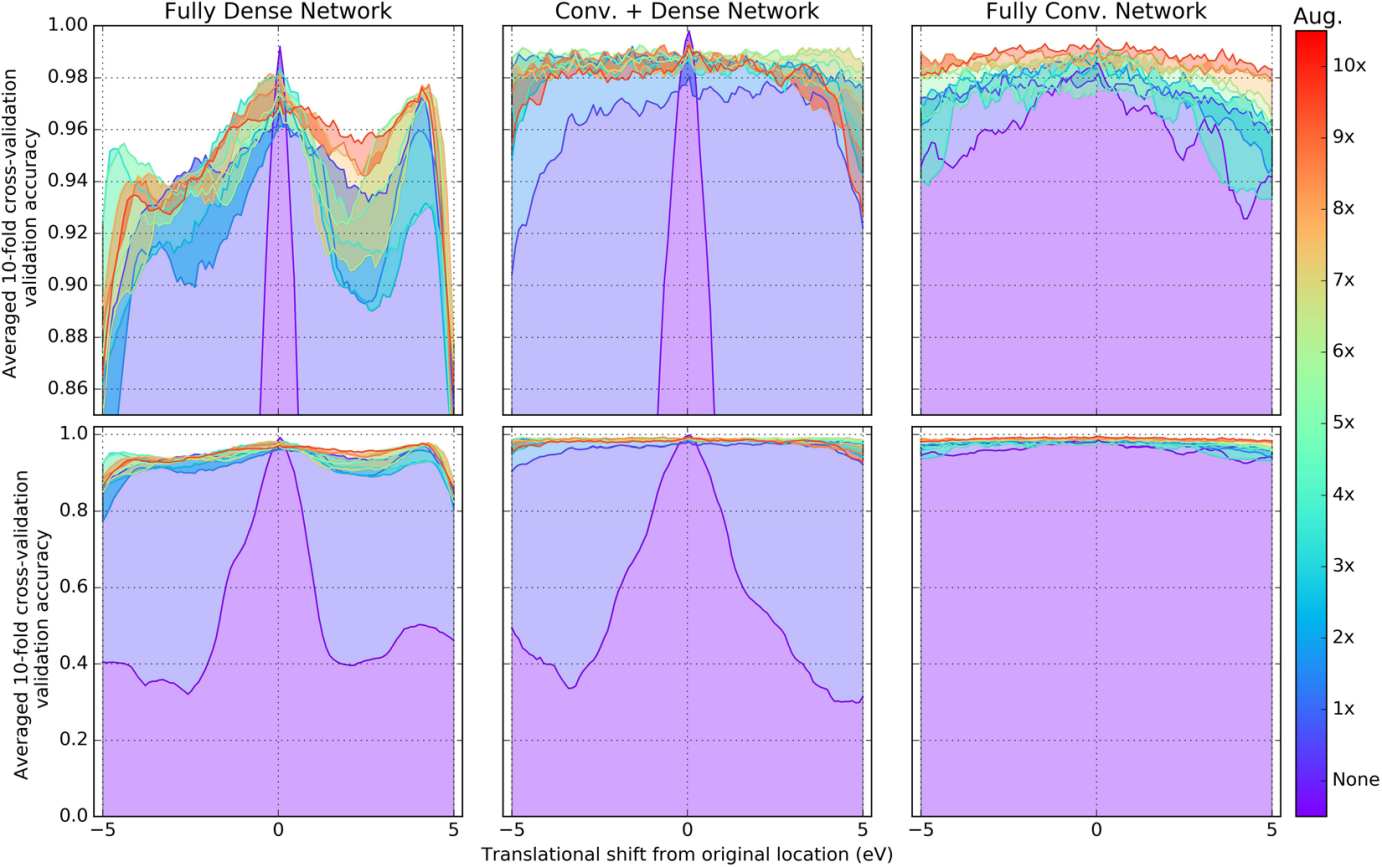
A comparison of how the validation-accuracy changes with respect to shifting the validation spectra for each neural network architecture and with varying degrees of training-set data augmentation. Training examples were randomly translated (−5 to +5 eV) in different scalar multiples of the training data and also compared against no augmentation at all. The upper row of plots contains a zoomed-in view from 85–100% validation accuracy while the lower row of plots contains the full range of accuracy for each respective style of neural network.

Translation-invariance was measured by cropping each validation example and moving the Mn ionization edge into different positions into the 300-length input vector. A 5 eV shift in this context is equivalent to moving the peak 50 bins out of the total 300 bins. To test translation-invariance, the validation-set spectra, for each cross-validation fold, were shifted left and right up to 5 eV and the resulting validation-accuracy was measured. To further probe if translation-invariance can be learned, data augmentation was used to randomly translate training examples between −5 and 5 eV. For each architecture, the training examples were randomly shifted (data augmentation) and this was performed for various scalar multiples of the training data ranging from 1–10x the original amount of training data. The results of the effect of translation-invariance with respect to neural network architecture and scalar multiples of training data can be found in Fig. 3.

As the amount of training data augmentation increases for the fully convolutional network, the performance increases (albeit subtly) (Fig. 3). This can be observed by looking at the gradient of color going from purple to red. However, this is not the case for the other two networks with no smooth increase in validation accuracy as the amount of augmentation increases.

Without applying translation data augmentation to the training set (dataset identified as “None”, dark purple colour in Fig. 3) and without shifting the validation data (i.e. zero-shift), all three networks are able to exceed 99% 10-fold cross-validation validation-set accuracy. When shifted, however, even without training data augmentation, the fully convolutional network is shown to produce a small decrease in validation accuracy as the spectra are translated, whereas the other two architectures have a sharp (<60%) decrease in accuracy near that of a random guess.

When applying translation data augmentation to the training set, the dense containing networks have a sharp increase in validation accuracy as the validation spectra are translated, suggesting that translation-invariance can be brute-force learned. When using the randomly shifted training data (“1× ” size, with random shifts) to train the dense containing networks, the fully convolutional network without any sort of data augmentation (dataset labeled as “None”) is still superior (this was measured by integrating the validation accuracy with respect to translation). It is only when large scalar multiples of training data are applied to the Conv. +Dense network that it is able to outperform the non-augmented fully convolutional architecture. However, when comparing the 10x trained fully convolutional network to 10x trained Conv. +Dense network, the fully convolutional network is still superior.

It is equally worth noting that the fully convolutional network is superior under these conditions despite containing only 60% as many weights as the convolutional and dense network, and only 6% as many weights as the fully dense network. The fully convolutional neural network is also much more constrained by using global average pooling as opposed to dense connections. This may aid in translation invariance. However, it was shown by Azulay and Weiss^18^ that popular pre-trained ImageNet classifiers (e.g. Inception^19^, VGGNet^13^) are not completely translation-invariant even when using global average pooling.

### Model Validation – Effect of noise

To test the robustness of convolutional neural networks to the noise frequently found in electron energy loss spectra acquired using transmission electron microscopes, a noise test was implemented using principal component analysis (PCA) for the fully convolutional neural network. PCA has grown increasingly popular at denoising EELS spectra recently^20–24^. Removing low variance principal components is effective at eliminating some types of noise frequently detected in EELS with minimal loss of signal (typically <10^−2^% of the signal is removed). Using these low variance principal components as a noise distribution, they were added to each input spectrum in scalar multiples ranging between zero and five times the baseline noise level. In this context, a scalar multiple of zero would be PCA cleaned data (removing low variance components), and a scalar multiple of 1 is the original signal.

**Figure 4:**
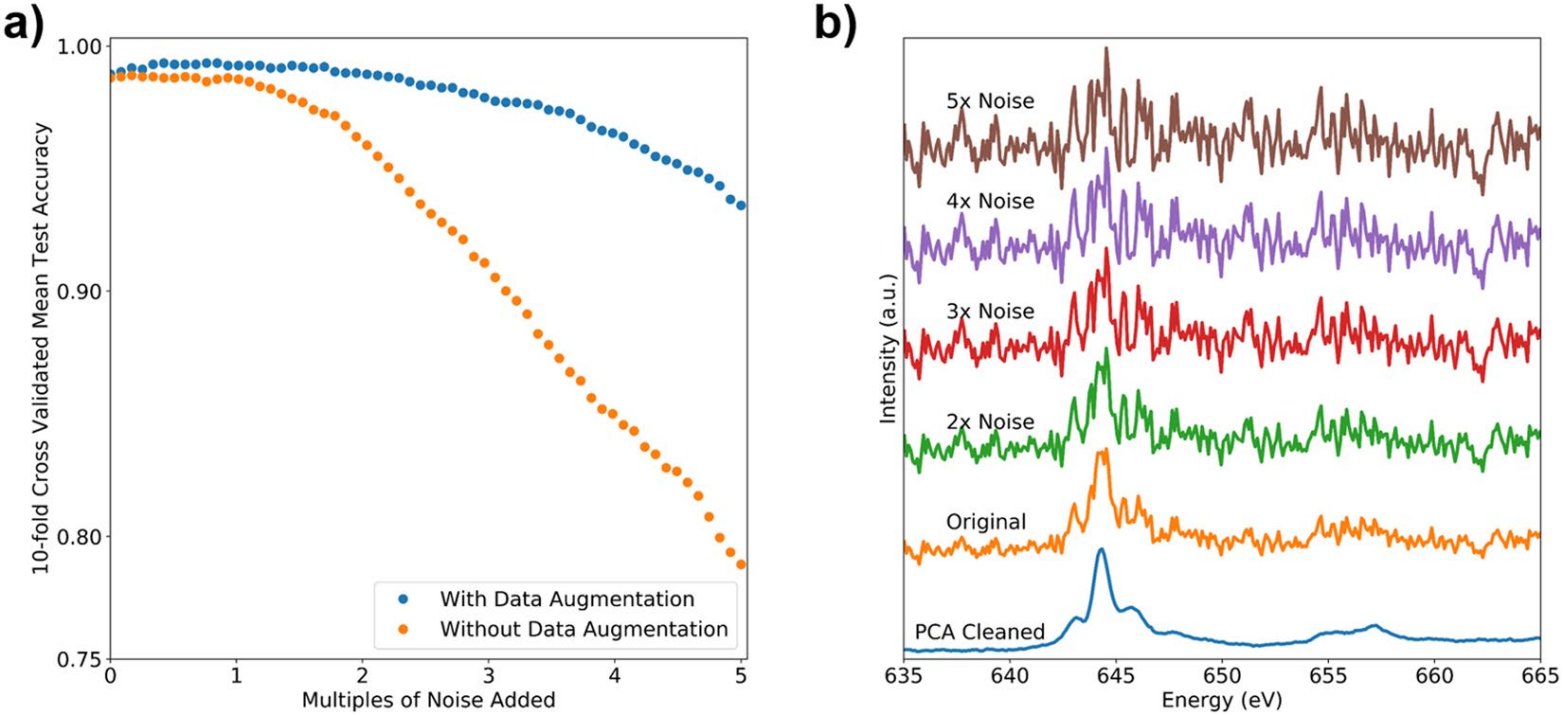
(**a**) A comparison of the effect of data augmentation on how the fully convolutional network when adding multiples of low variance principal components. The x-axis refers to multiples of noise added to the validation-set. With and without data augmentation refers to adding noise to the training data. (**b**) An example of spectra showing the effect of scalar multiples of low variance principal components added on to spectra.

PCA was performed on each validation-set during cross-validation and low variance principal components were calculated on the validation-set and added back to the validation data in different scalar multiples. This measures how well the classifier can predict the oxidation state in the presence of extreme noise that a trained human spectroscopist would have great difficulty in classifying. As an example of what the signal-to-noise ratio looks like qualitatively, the effect of different scalar multiples of low variance principal components on spectra is shown Fig. 4b.

An additional test was also performed to see if the model could be made robust to high levels of noise by deliberating adding noise to the training data. This is called training data augmentation. PCA was performed on every training fold during cross-validation and low variance principal components were added to each training example. The neural network was then evaluated using the data augmented folds of the validation-set to measure how the validation accuracy changes with increasing validation-set noise. The comparison between the fully convolutional classifier trained with or without training data augmentation is shown in Fig. 4a.

This test shows that, in the presence of noise that is a 5x scalar multiple of low variance principal components, the data augmented classifier is able to exceed 93% validation-set accuracy. Without data augmentation the accuracy decreases moderately as noise is added with a validation-set accuracy of 78% at 5x the base-line noise level. It should be noted that a 1x scalar multiple of the low variance principal components is indeed just the original signal. A 0x scalar multiple in this context is a PCA-cleaned spectrum with low variance components being removed.

### Model Testing - Digitized Reference Spectra

The three neural networks were tested against the 31 digitized spectra taken from the publications by Zhang *et al*., Tan *et al*., and Garvie *et al*.^8,9,16^ to probe generalizability in the presence of different instruments, calibration, and resolution. This dataset contains 12 Mn^2+^ spectra (tetrahedral, octahedral, and dodecahedral coordination), 10 Mn^3+^ spectra (octahedral), and 9 Mn^4+^ spectra (octahedral). These spectra are also shifted significantly (~3 eV) compared to the acquired spectra, likely the result of different instrument calibration, and have different levels of noise (Fig. 2).

The three architectures tested in this study tested against the 31 digitized reference spectra. The test accuracies on the digitized reference spectra dataset are shown in Table 1.

**Table 1:** Performance (validation accuracy) of each neural network architecture on the digitized reference spectra dataset. Each network is also compared when using translation data augmentation during test time in different scalar multiples and also without any augmentation.

Classifier	No Translation Augmentation	Random Shifts (1x data)	Random Shifts (10x data)
Fully Conv.	1.00	1.00	1.00
Fully Dense	0.613	1.00	1.00
Conv. + Dense	0.742	1.00	1.00

In analyzing Table 1, the fully convolutional neural network is proven to be extremely successful on the digitized reference spectra dataset even without data augmentation. In contrast, dense layer containing architectures fail to generalize to data outside of their training distributions and incorrectly classify Mn^2+^ and Mn^4+^ when data augmentation is not used. These results agree with the translation-invariance test in Fig. 3 that the dense layer containing networks have difficulty performing classification on datasets too dissimilar from their training data since the digitized reference spectra are shifted significantly (Fig. 2). It is only with data augmentation that the dense containing networks are accurate.

Neural networks are apparently capable of classifying Mn compounds that do not have the same coordination (octahedral) as the acquired training data such as tetrahedral and dodecahedral compounds. This is due to the fact that, as demonstrated by Garvie *et al*. and Tan *et al*., there are strong similarities in the shape of the fine-structure of the core-loss edges between various Mn, Fe and V compounds of the same valence^9,16^ for different coordinations, at least at the energy resolution used for the experiments.

The activations for each layer of the fully convolutional neural network on the digitized reference spectra dataset can be found in the Supporting Information.

### Visualizing the feature space using t-SNE

**Figure 5:**
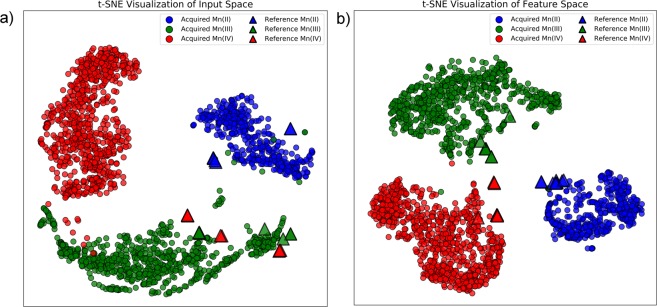
2D t-SNE visualization of the 300-dimensional input space (**a**) and of the 72-dimensional feature space of the trained fully convolutional network (**b**). The manifold of the feature space brings the digitized reference spectra (triangles) closer to the clusters of the acquired data as opposed to the original input space.

To help demonstrate the success of the fully convolutional network on the digitized reference spectra test-set in the absence of training data augmentation, the 300-length preprocessed spectra (both acquired and also the reference spectra) were projected onto a 2D plane using a t-distributed stochastic neighbor embedding (t-SNE) technique. t-SNE developed by van der Maaten and Hinton^25^ is a popular non-linear dimensionality reduction technique which attempts to preserve the distribution of clusters in the original high-dimensional space when projecting the data onto a 2D plane for visualization purposes. Note that the analysis presented here is entirely qualitative to aid in exploratory visualization. The visualizations shown in Fig. 5 were initialized with PCA, have a perplexity of 100, and were run for 10 000 iterations. A variety of visualizations with perplexities between 20 and 100 can be found in the Supporting Information.

The features produced by the final feature extraction layer prior to global average pooling of the fully convolutional network, consisting of 12 vectors of length 6, was flattened into a 72-length vector and also visualized using t-SNE.

The classes can be easily differentiated on the feature-space shown in Fig. 5b. In the feature-space, it is evident that the reference spectra (the triangles in Fig. 5) are contained within the clusters of acquired spectra. This is in contrast to the t-SNE visualization of the input space (Fig. 5a) where all Mn^4+^ are incorrectly classified as Mn^3+^.

The digitized reference spectra dataset is very different from the acquired dataset (cleaner signal and shifted 3 eV). It is evident from the t-SNE visualization of the full input spectra space that these two datasets lie in different distributions. We can gauge this qualitatively by observing that reference Mn^4+^ are more closely similar to acquired Mn^3+^ than they are to acquired Mn^4+^.

## Discussion

In the field of electron energy loss spectroscopy (EELS), spectrometer calibration is often very difficult and leads to non-reproducible data between research groups. In addition to this, the noise profile is very different between instruments so, typically, dimensionality reduction (unsupervised learning methods or manual feature extraction) of the data needs to be performed to compare results between instruments. In this study, we create a new method of spectroscopy analysis using deep convolutional neural networks. These networks are proven to have significant advantages over all other methods in EELS analysis. In addition, our proposed fully convolutional neural network also has significant advantages over other neural networks used in the chemometrics literature with respect to translation-invariance. Our convolutional neural network is immune to calibration differences and have high noise tolerances which exceeds the ability of the currently used methods.

Convolutional neural networks are automated feature extractors. They require no domain knowledge, and require no manual feature collection, such as taking the FWHM of peaks or determining peak on-set. It is clear from the t-SNE visualization in Fig. 5 that the feature “fingerprints” produced by neural networks successfully embed discriminating differences between the three oxidation state clusters from both reference data digitized from publications and spectra that we acquired. The digitized reference spectra dataset is very different from the acquired dataset (cleaner signal and shifted 3 eV) and it is evident from the t-SNE visualization of the full input spectra space that these two datasets lie in different distributions. Despite this, however, our fully convolutional neural network is proven to successfully perform classification.

## Conclusions

We have demonstrated that traditional neural architectures, such as densely connected neural networks, or convolutional neural network feature extractors attached to a densely connected neural network, have limited translation-invariance for energy-series classification tasks. In contrast, our developed fully convolutional neural network uses global average pooling on the final feature layer and retains translation-invariance. Translation-invariance is particularly important in spectroscopy because a practical classifier would be one trained from one data set acquired from one spectrometer but potentially tested on another instrument with different calibration or different sessions where stability of the instrument is not perfectly controlled. Therefore, fully convolutional classifiers have the potential to remove some qualitative methodologies from spectroscopy interpretation and can be extended to additional classes (valences, elements, bonding states etc.) provided there is training data. These classifiers may be able to be applied to many other spectroscopic methods where discrimination between spectra is determined by peak shape, instances with high noise, or cases where calibration is difficult and unreliable over time.

## Methods

### Architecture – Fully Dense Network

The densely connected neural network receives the (300,1) length input spectra and passes it through two layers of 32 hidden units each. Each hidden unit uses a rectified linear unit (ReLU) activation function. Every layer uses batch-normalization after activation and has a 50% neuronal dropout in between layers to limit over-fitting.

To re-normalize the data between hidden layers, a per-batch normalization is performed to mean center the data and to standardize the data to unit variance. The data is originally preprocessed to be normalized before model training. However, after subsequent affine transformations and activations, this will no longer be the case. This phenomenon is called the internal covariate shift and this batch normalization procedure developed by Ioffe and Szegedy is a commonly used method for normalization between operations in many popular neural network architectures^11^.

Overfitting occurs when the network has learned to memorize the training distribution and no longer can generalize to the validation or test distributions. One commonly used technique to limit large amounts of overfitting is to use the stochastic method of neuron dropout developed by Srivastava *et al*.^10^. Dropout is used to prevent overfitting by randomly selecting a percentage of weights per batch between the weights connecting the feature extractor neurons and the classifier neurons and setting them equal to zero. Dropout is used so that, during model training, the optimum parameter configuration will be one that does not heavily rely on a small subset of parameters.

The last hidden layer is connected to three neurons (one for each class of Mn) and activated via a softmax function (i.e. multinomial logistic regression) to draw a decision boundary to determine which class is likely present.

The predicted class probability $p({y}_{k}|z,\theta )$ of the correct label *y*_*k*_ given a set of parameters *θ* with an input spectrum feature vector *z* is shown below. This softmax function is the final layer in all of the neural architectures studied here and is a commonly used classification operation used in neural networks.

$$p({y}_{k}|z,\theta )=\frac{{e}^{{z}_{j}}}{{\sum }_{k=1}^{K}{e}^{{z}_{k}}}$$

### Architecture – Convolutional and Dense Network

The second network can be considered to have two parts. The first part is used to perform automated feature extraction by using a convolutional neural network. The convolutional neural network is used to project the spectra onto a vector space that more clearly encodes discriminating differences between the classes. The second part is a densely connected neural network which uses these features to perform classification and to draw a decision boundary.

The feature extraction architecture is composed of 5 layers. Each feature extraction layer has first a 1D convolution, activation using a rectified linear unit (ReLU), followed by batch-normalization, then finally an average pooling layer (i.e. 2x averaged down-sampling).

Formally, the convolution in a convolutional graph network can be written as follows,

$${z}_{j}^{(L+1)}=(\sum _{k}{w}_{j}\,\ast \,{a}_{k}^{(L)})+{b}_{j}$$

where ${z}_{j}^{(L+1)}$ is the output in the hidden layer (*L* + 1) at neuron index *j* of that layer, receiving inputs from previous layer neurons indexed by *k*. The convolution kernel (also known as a filter) *w*_*j*_ is of fixed length and slides across the previous layer activations ${a}_{k}^{(L)}$ at a stride of 1 to perform a sliding dot product. This is summed across all layer (*L*) neurons indexed by *k* before a bias term *b*_*j*_ is added.

The input into each feature-extraction layer is convolved with a sliding (stride of 1) kernel of length 9, 7, 7, 5, or 3 for the 1^st^ through 5^th^ blocks respectively. The number of filters per block gradually expands from 2, 2, 4, 8 and 12 filters for the 1^st^ through 5^th^ blocks respectively. The premise behind architecture construction was to start with a large number of filters similar to VGG16 (which contains millions of free parameters), and shrink the network until validation and test set accuracies started significantly falling.

The output shape after the 5^th^ feature extraction layer is 12 activations of size (6,1). These features are then flattened to (72,1) before being put into a dense neural network to perform classification.

### Architecture – Fully Convolutional Neural Network

The most successful architecture studied here was the fully convolutional architecture which avoids using dense layers. It is comprised of the same feature extraction architecture as the network above to produce 12 vectors of size (6,1) as features. To perform classification on these features, a classification architecture was created similar to the dense-layer free architecture which have been used in recent architectures such as MobileNet, and SqueezeNet^14,15^. This style of classification architecture consists of: dropout, convolution (without non-linear activation), global average pooling and activation via softmax. A dropout rate of 0.8 (determined via 10-fold cross validation) was used to enforce a strict information bottleneck.

Three convolution filters (one per class) with a stride of 1 and kernel size of 1 are used to create a shape of 3 vectors of size (6,1). Each of the three vectors is then averaged into 1 number (i.e. global average pooling) to produce 1 number per filter. This value is then passed into the logistic regressor (softmax) function to convert it into predicted class probabilities to perform classification.

### Computational Hardware and Software

Model training occurred on a GPU (GTX 1060, 6 GB video card RAM) and it took 3 seconds per epoch for 100 000 data augmented training spectra to be passed forward and backwards through the graph in batches of 2048. All neural network training was performed using Tensorflow and Keras in Python. A full description of dependencies and the training and testing scripts can be found in this repository on GitHub (https://github.com/MichaelChatzidakis/Mn_Classifier_CNNs). Data is available upon request.

### Preprocessing

Every spectrum image was unzipped and appended to the same array per valence state using the Python library *HyperSpy* to open the *Digital Micrograph* .dm3 files. The EELS spectra were then spectrally cropped to a length of 300 histogram bins (dispersion of 0.1 eV/channel) near the Mn L_2,3_ core-loss edges so that only Mn L_2,3_ edge data was included. There were a total of 1273, 1342 and 1687 spectra for Mn^2+^, Mn^3+^ and Mn^4+^ respectively.

Dark-Field images corresponding to the Spectrum Images can be found in the Supporting Information. Each image was taken over a Mn oxide particle, so part of most images are solely the supporting substrate and not just the Mn oxide in question. To remove pixels containing only substrate signals from the coalesced dataset, k-Means clustering was performed on each class of the data (Mn^2+^, Mn^3+^ and Mn^4+^). By qualitatively inspecting the cluster center, it is apparent which cluster center belongs to the Mn L_2,3_ core-loss edges and which cluster center belongs to the substrate (where no Mn L_2,3_ edge is present). The cluster centers are shown in the Supporting Information.

In electron energy loss spectroscopy, background removal is required to isolate only one core-loss ionization edge. It is a common practice to fit a power-law spectrum to the points before the edge in question. To remove the background prior to the onset of the core-loss edge, a generalized power-law was least-squares fitted to the indices (50 bins) prior to the edge onset and subtracted from each spectrum. Next, each spectrum was individually normalized between 0.0 and 1.0 and mean-subtracted. The choice of these preprocessing steps was empirical and determined via cross-validation.

There is a sizeable portion of low-quality data present in the dataset as a result of trying to get a wide range of varying thickness and crystallographic orientations in the samples during acquisition. If samples are too thick then they are not electron transparent and no ionization edge is visible. Typically, only samples under 100 nm are thin enough for the beam to transmit through. Many of the Mn oxide crystals which we acquired data from are “wedge” shaped, so only a portion of the collected data is actually thin and electron transparent. To remove these low-quality spectra, an iterative k-Means clustering approach was used. Each class of Mn was clustered with two or three cluster centers and, through qualitative inspection of the cluster center mean, thick and thin samples could be discriminated by simply seeing if the peaks were broadened compared to the literature.

### Sample Preparation and Microscope Parameters

All samples were acquired using a monochromated FEI Titan 80–300 transmission electron microscope (TEM) operating at an accelerating voltage of 80 keV. The FWHM of the zero-loss peak was ~0.2 eV. Reference Mn oxide samples above 99% purity (obtained from Sigma-Aldrich) were crushed between SiO_2_ glass slides to produce a fine nanoparticulate powder. A holey carbon TEM grid was tapped onto the glass slide to adsorb fine particulates. In STEM mode, spectrum-images were collected over a 2D area on the edges of the nanoparticulate Mn oxides to capture the thinnest areas. 10, 10 and 15 spectrum images of each of the MnO, Mn_2_O_3_ and MnO_2_ powder samples were acquired for a total of 1604, 1512 and 1863 individual spectra respectively. Spectrum images were acquired on a variety of thicknesses and crystallographic orientations. See the Supporting Information for annular dark-field images of a selection of spectrum images.

## Supplementary information

Supplementary Information
