# Supplementary material for: Towards calibration-invariant spectroscopy using deep learning
Source: Sci Rep. 2019 Feb 14;9:2126. doi: 10.1038/s41598-019-38482-1 (PMC6376024; doi:10.1038/s41598-019-38482-1)
Supplement: Supplementary file 1 — Supplementary Information [file 41598_2019_38482_MOESM1_ESM.pdf]

Towards calibration-invariant spectroscopy using deep learning

M. Chatzidakis[1,2] , G. A. Botton[1,2]

[1]Department of Materials Science and Engineering, McMaster University, Hamilton, ON, L9H 4L7 Canada,

[2]Canadian Center for Electron Microscopy, Hamilton, ON, L8S 4M1, Canada

S1.0 – Supporting Figures

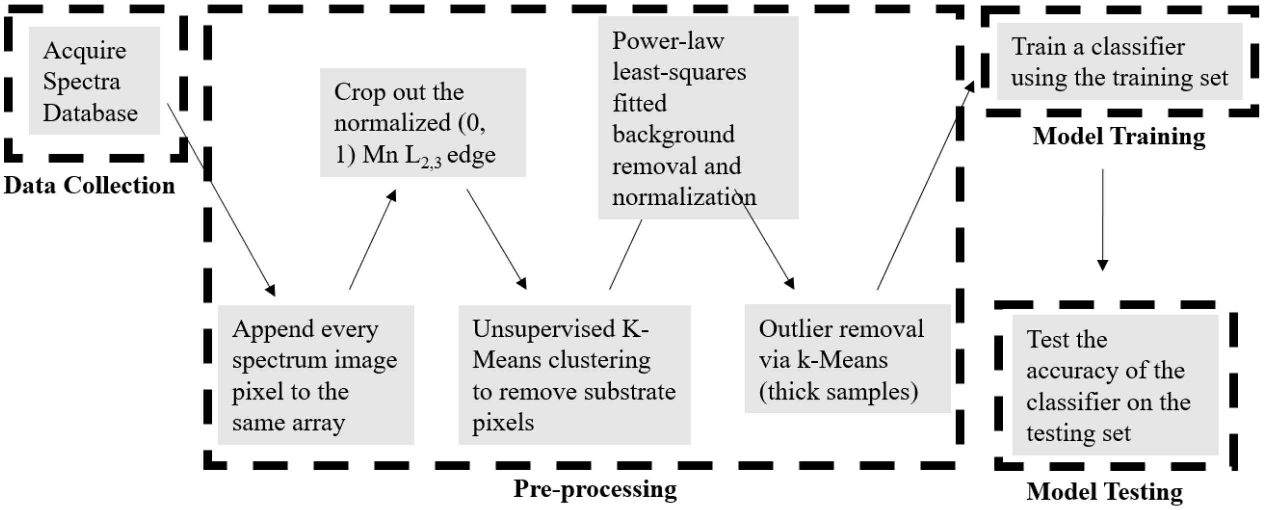

Figure S1. Work-flow of going from data collection to model testing and all of the steps in between.

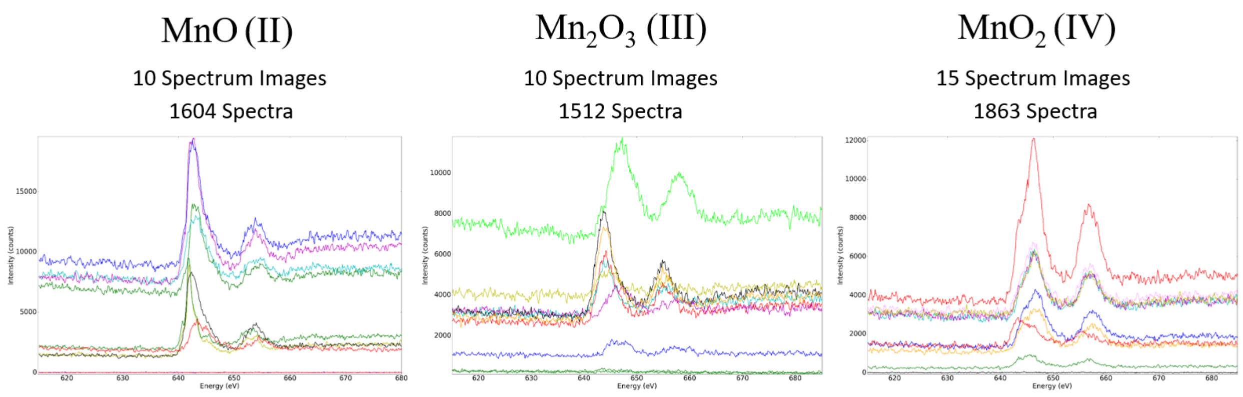

Figure S2. Raw spectra for each of the three valences of Mn.

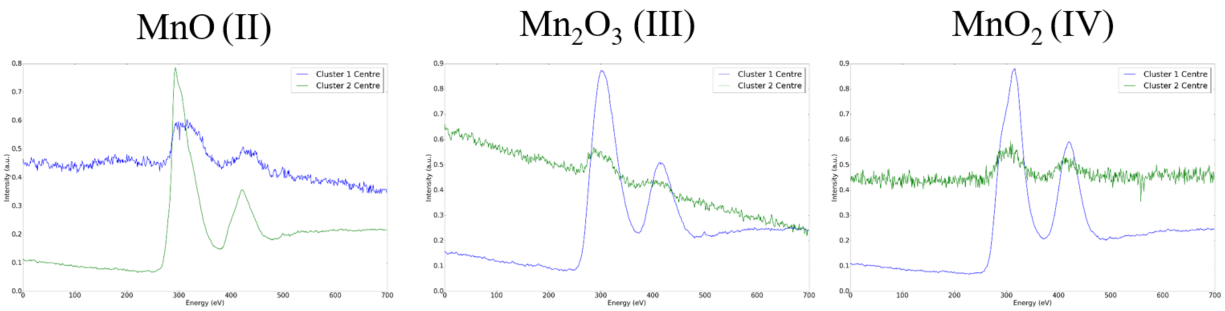

Figure S3. Example of K-means clustering on each Mn oxide sample assuming two cluster centers. It is obvious qualitatively which cluster the substrate belongs to.

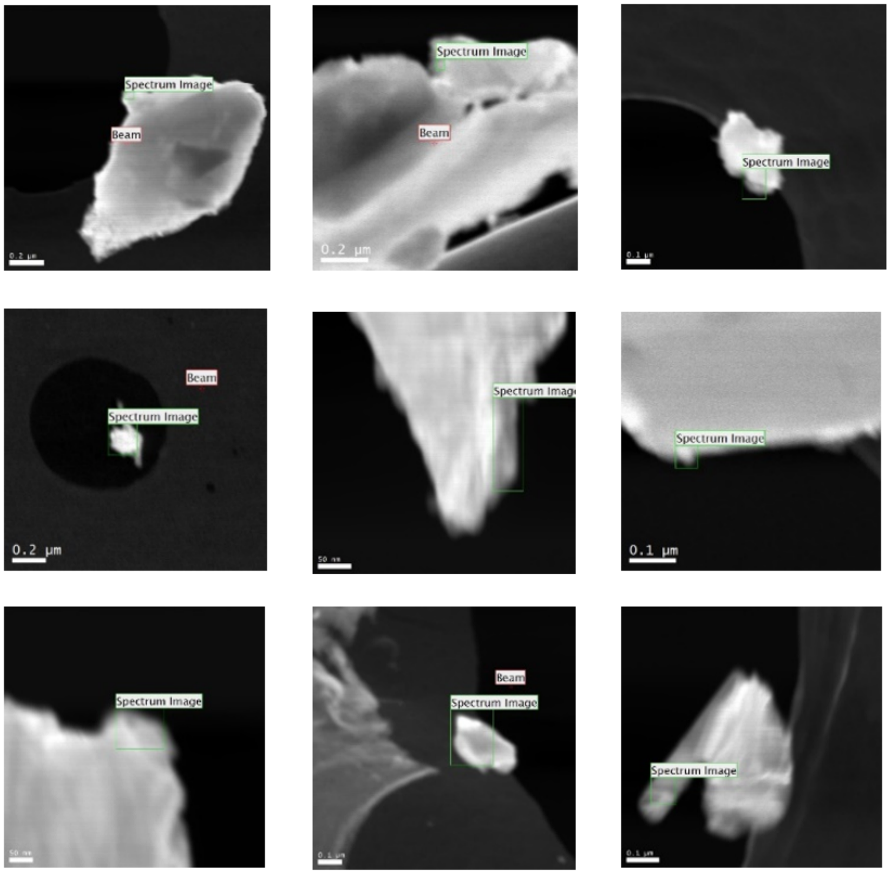

Figure S4. Annular Dark-Field (ADF) images of some of the samples showing varying sizes of Mn oxide nanocrystals. Acquisitions were carried out on the edges of most samples to get spectra from thin areas.

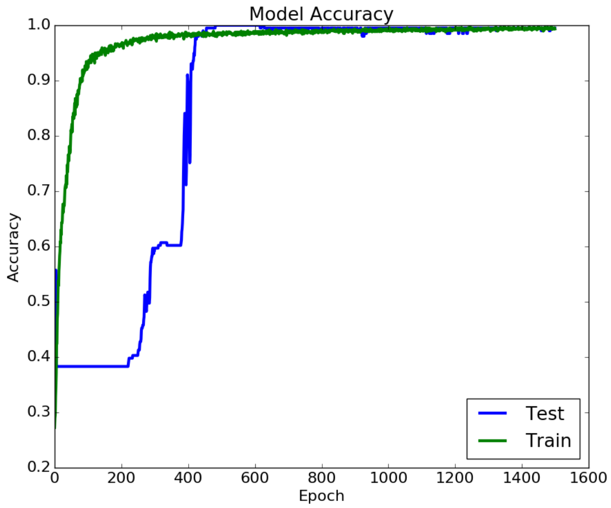

Figure S5. Validation accuracy of one fold for the fully convolutional neural network.

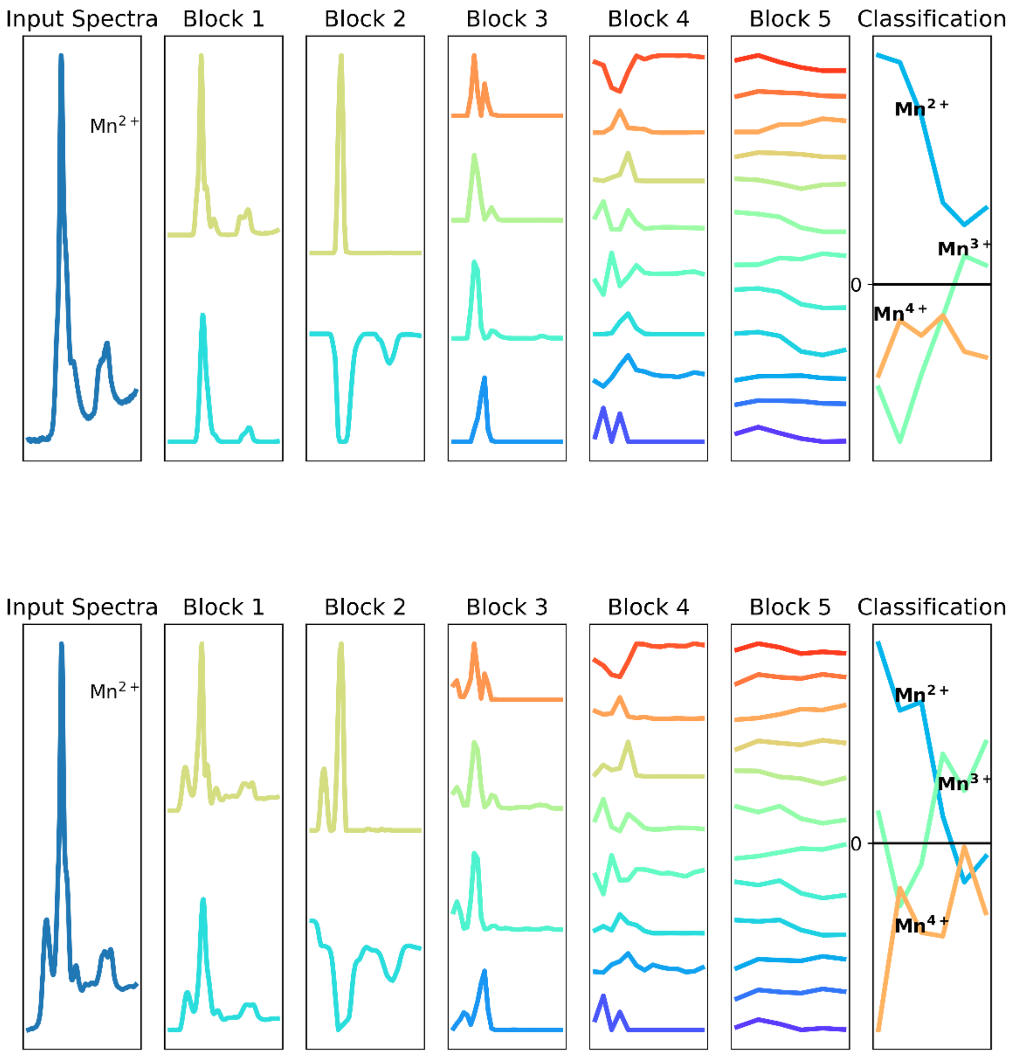

Figure S6. (top) Intermediate activations averaged over all acquired Mn^{2+} spectra. (bottom) Intermediate activations averaged over all digitized reference Mn^{2+} spectra.

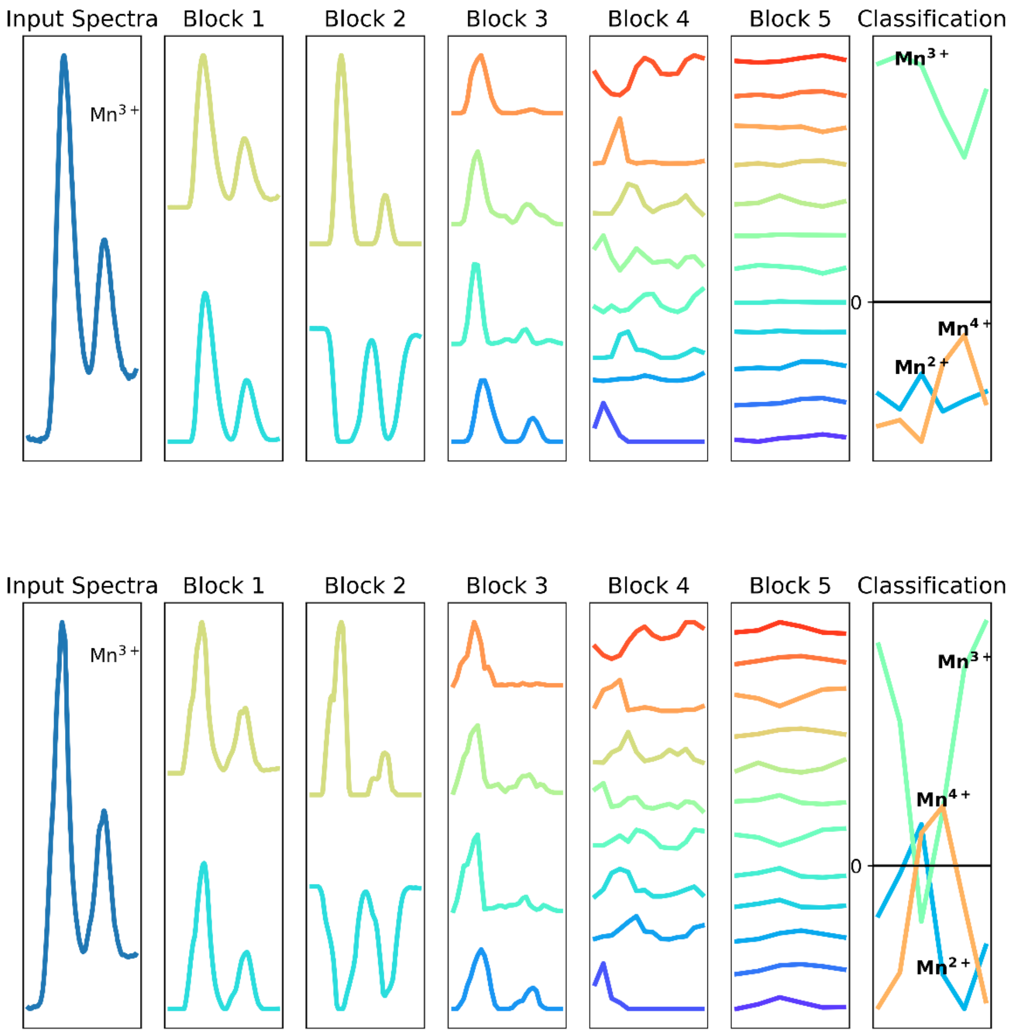

Figure S7. (top) Intermediate activations averaged over all acquired Mn^{3+} spectra. (bottom) Intermediate activations averaged over all digitized reference Mn^{3+} spectra.

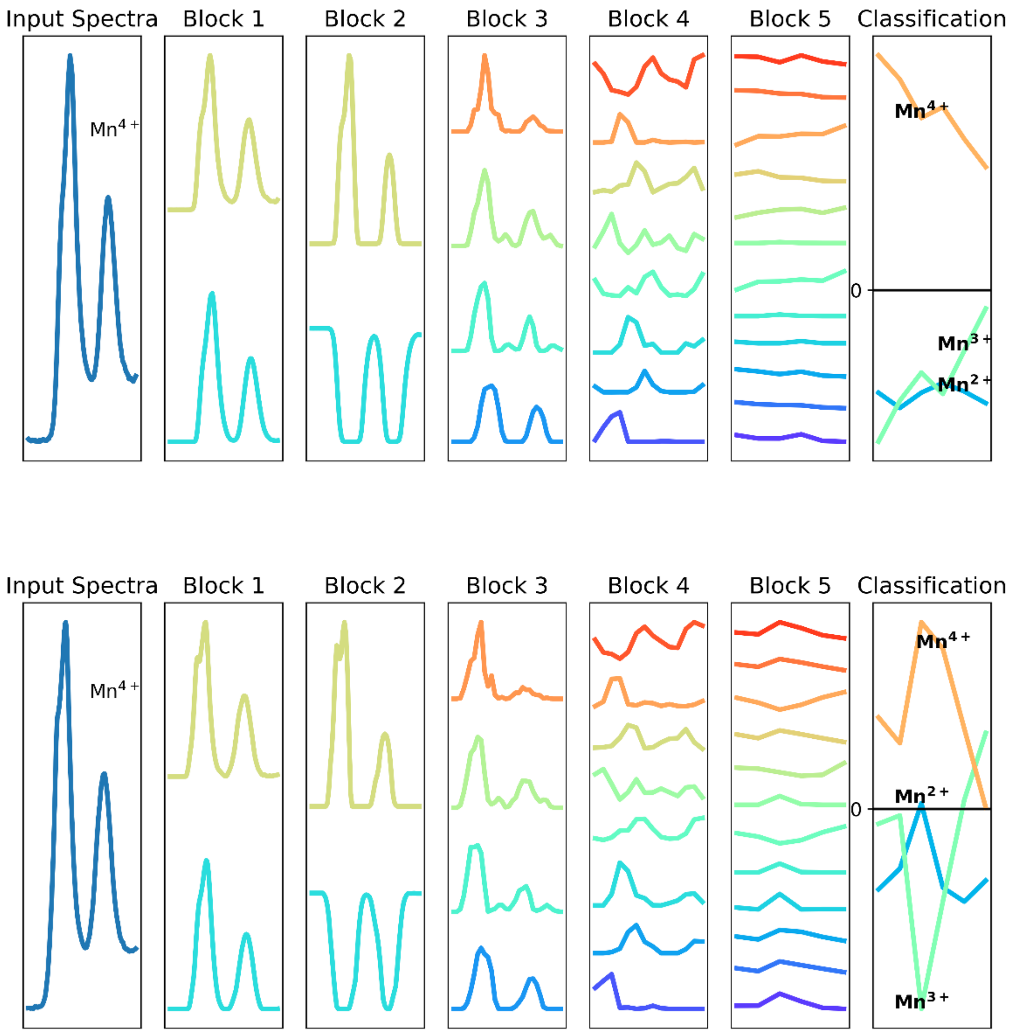

Figure S8. (top) Intermediate activations averaged over all acquired Mn^{4+} spectra. (bottom) Intermediate activations averaged over all digitized reference Mn^{4+} spectra.

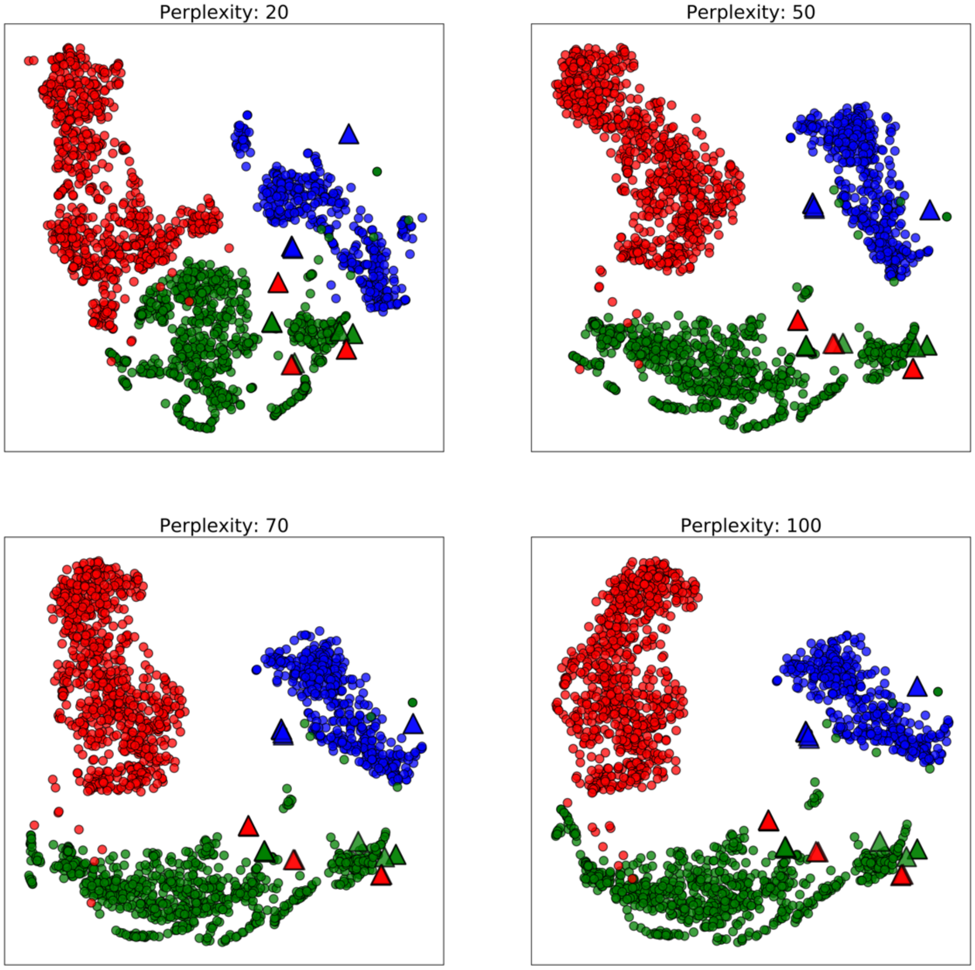

Figure S9. t-SNE of the input spectra space across many values of perplexity. Circles represent acquired data whereas triangles are digitized reference spectra. Blue, green and red represent Mn^{2+}, Mn^{3+}, and Mn^{4+} respectively.

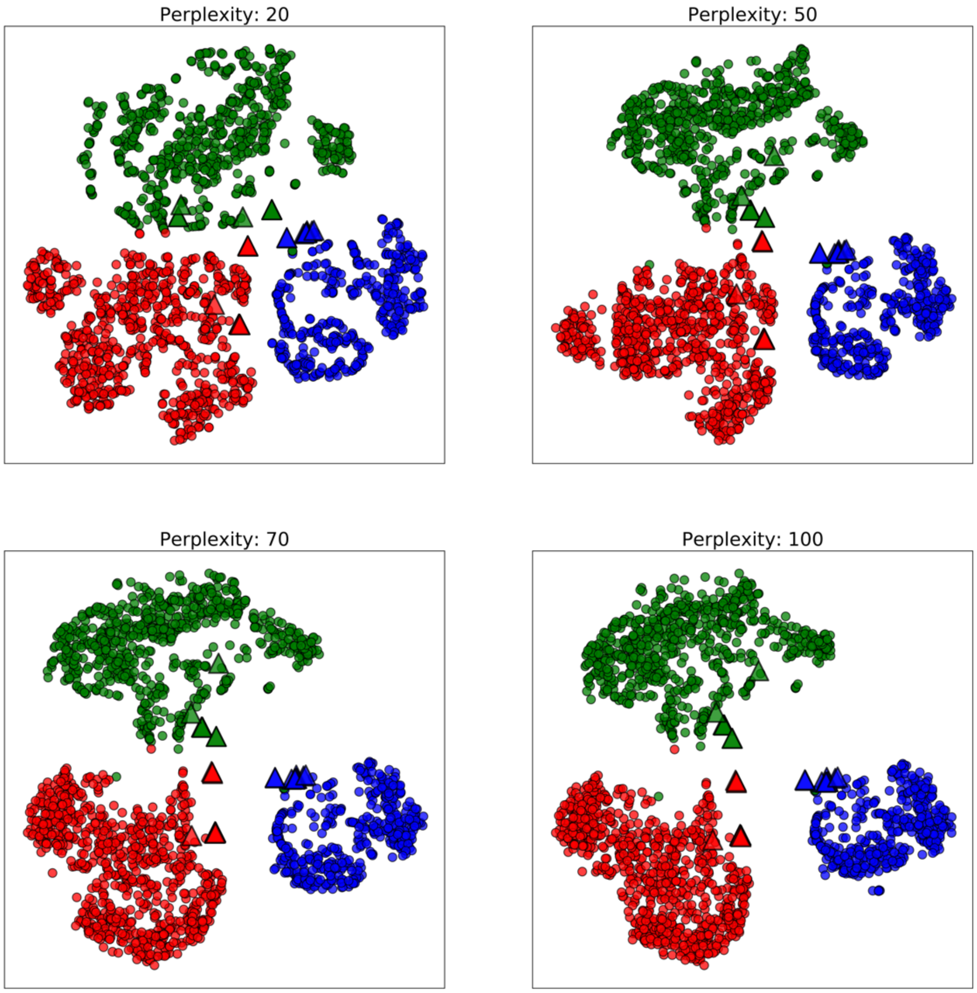

Figure S10. t-SNE of the feature space across many values of perplexity. Circles represent acquired data whereas triangles are digitized reference spectra. Blue, green and red represent Mn^{2+}, Mn^{3+}, and Mn^{4+} respectively.
